# An In Silico Model for Predicting Drug-Induced Hepatotoxicity

**DOI:** 10.3390/ijms20081897

**Published:** 2019-04-17

**Authors:** Shuaibing He, Tianyuan Ye, Ruiying Wang, Chenyang Zhang, Xuelian Zhang, Guibo Sun, Xiaobo Sun

**Affiliations:** 1 Beijing Key Laboratory of Innovative Drug Discovery of Traditional Chinese Medicine (Natural Medicine) and Translational Medicine, Institute of Medicinal Plant Development, Peking Union Medical College and Chinese Academy of Medical Sciences, Beijing 100193, China; wenyuxuan2530@163.com (S.H.); yetianyuan2013@163.com (T.Y.); wangruiying8866@163.com (R.W.); zhangchenyang0120@126.com (C.Z.); zxl2022@126.com (X.Z.); 2 Key Laboratory of Bioactive Substances and Resource Utilization of Chinese Herbal Medicine, Ministry of Education, Beijing 100193, China; 3 Key Laboratory of Efficacy Evaluation of Chinese Medicine against Glycolipid Metabolic Disorders, State Administration of Traditional Chinese Medicine, Beijing 100193, China; 4 Key Laboratory of New Drug Discovery Based on Classic Chinese Medicine Prescription, Chinese Academy of Medical Sciences, Beijing 100193, China

**Keywords:** DILI, hepatotoxicity, in silico, machine learning, molecular descriptors

## Abstract

As one of the leading causes of drug failure in clinical trials, drug-induced liver injury (DILI) seriously impeded the development of new drugs. Assessing the DILI risk of drug candidates in advance has been considered as an effective strategy to decrease the rate of attrition in drug discovery. Recently, there have been continuous attempts in the prediction of DILI. However, it indeed remains a huge challenge to predict DILI successfully. There is an urgent need to develop a quantitative structure–activity relationship (QSAR) model for predicting DILI with satisfactory performance. In this work, we reported a high-quality QSAR model for predicting the DILI risk of xenobiotics by incorporating the use of eight effective classifiers and molecular descriptors provided by Marvin. In model development, a large-scale and diverse dataset consisting of 1254 compounds for DILI was built through a comprehensive literature retrieval. The optimal model was attained by an ensemble method, averaging the probabilities from eight classifiers, with accuracy (ACC) of 0.783, sensitivity (SE) of 0.818, specificity (SP) of 0.748, and area under the receiver operating characteristic curve (AUC) of 0.859. For further validation, three external test sets and a large negative dataset were utilized. Consequently, both the internal and external validation indicated that our model outperformed prior studies significantly. Data provided by the current study will also be a valuable source for modeling/data mining in the future.

## 1. Introduction

As the first organ that comes into contact with most of the products of digestion, the liver plays a critical role in energy exchanges and the biotransformation of xenobiotics. Livers suffering from damage always disrupt the normal metabolism, and even lead to liver failure [1,2,3]. During the past decades, as one of the major types of liver damage, drug-induced liver injury (DILI) continues to be an active area of research. In the process of drug discovery, DILI has been identified as the most frequent cause for the termination of drug development programs. Additionally, hundreds of drugs have been withdrawn from the market and rejected in the new drug applications for the evidence of liver injury in the past half century [4,5,6,7]. Elimination of drug candidates with DILI risks early in drug discovery may be an effective strategy to reduce the rate of attrition and decrease the cost of drug discovery. Therefore, research that attempts to assess the DILI risk of drugs and drug candidates should be given more attention.

Traditionally, the hepatotoxicity of drugs was detected experimentally. However, one cannot deny that many experiment methods are time-consuming and labor-intensive. In addition, DILI caused by most drugs is of an idiosyncratic nature and usually cannot be identified by the regulatory required animal/cell toxicity experiments [8,9,10]. Compared to detecting hepatotoxicity experimentally, predicting the DILI risk by in silico models is more time-saving and low-cost, and is considered to be effective in assessing the potential DILI risk of drug candidates [11,12].

Recently, a large number of in silico approaches to DILI prediction have been reported, which can be categorized into two main groups: statistical-based and expert-based approaches [13,14]. Statistical-based approaches usually attempt to correlate molecular descriptors or molecular fingerprints with DILI outcome by machine learning methods [15]. The extraction of structural alerts always is the first step to creating expert-based models. Thereafter, relationships between the structural alerts and the biological activity were defined based on the researcher’s own understanding of the toxicological mechanisms [16]. Actually, structural alerts indeed lead to a deeper insight into the toxicological mechanisms of drugs. However, Quantitative structure–activity relationship (QSAR) models developed based on structural alerts always failed to provide satisfactory predictability [17,18,19,20]. Additionally, some researchers have pointed out that structural alerts are not effective predictors of toxicity [21,22]. Compared to expert-based approaches, statistical-based approaches seemed to be more effective, and gained increasing attention in recent years. In 2010, Ekins et al. [23] established an in silico model for predicting DILI based on a dataset consisting of 295 unique compounds, and a test set containing 237 compounds was used to perform the external validation. As a result, both the internal and external validation yielded accuracy values of about 60%. Currently, there have been numerous statistical-based models reported. However, they rarely provided satisfactory predictability. Although several models showed relatively higher accuracy, they always suffered from imbalanced performance or small datasets [1,24,25,26,27,28]. Actually, imbalanced performance and limited dataset almost were the common deficiencies of all the QSAR models available in the literature. To our knowledge, there were only nine papers that reported QSAR models for DILI risk assessment based on large-scale datasets (sample size >1000). However, these models either suffered from imbalanced performance [29,30] or failed to provide satisfactory predictability [31,32,33,34,35]. Based on a large-scale data set consisting of 3712 compounds, a total of 21 QSAR models were established by Mulliner et al. of which the area under the receiver operating characteristic curve (AUC) values ranged from 0.71 to 0.75 [36]. By employing 3 machine learning algorithms and 12 molecular fingerprints, Ai et al. [37] developed an ensemble model based on 1254 diverse compounds, and achieved an average ACC of 71.1 ± 2.6% and AUC of 0.764 ± 0.026 within five-fold cross-validation. As we know, this may be the most recent QSAR model for predicting DILI. Taken together, models proposed by prior studies are still not sufficiently predictive, and there has been considerable room for improvement in DILI prediction.

A large-scale and balanced dataset always not only benefits acquiring sufficient coverage of the chemical space of interest but also assists in decreasing the risks of imbalance of models. Therefore, a fundamental part of this endeavor was to build an extensive and diverse dataset of DILI through a system literature retrieval and a critical data-filtering strategy. To get a balanced training set, the Kennard–Stone algorithm was used to extract the non-DILI data. For each compound in the training set, a set of molecular features, 29 physicochemical properties and topological geometry properties (56), were calculated and the nonredundant features were utilized to train classifiers. A total of eight effective and widely used machine learning algorithms were involved, including Naïve Bayes, K-nearest neighbor, Kstar, AdaBoostM1, Bagging, J48, Random Forest, and Deeplearning4j. By averaging the probability from each base classifier mentioned above, we also attempted to generate an ensemble model. For validation, three external test sets and a negative dataset were utilized to make comparisons between our model and prior studies.

## 2. Results

### 2.1. Optimizing the Training Set

After data filtering, the final dataset consisted of 1416 unique compounds (707 positives, 709 negatives). As expected, the initial modeling attempts failed to yield models with satisfactory performance. We thought such a situation may be attributed to three aspects as follows. Firstly, the whole dataset included 1416 unique compounds. To our knowledge, this is the largest dataset for hepatotoxicity. So large a sample size increased the diversity and complexity of the data structure. Secondly, although we had filtered the data based on a complex workflow, there still might have existed some poor data in the dataset, including false negatives and false positives from the literature. Thirdly, the existence of outliers also brought noise to the development of models. To further improve the data quality, a voting method was applied to filter the dataset. Initially, eight effective classifiers, including Naïve Bayes, KNN, Kstar, AdaBoostM1, Bagging, J48, Random Forest, and Dl4j, were applied to train the dataset within 10-fold cross-validation. For each compound, the predicted result returned by each classifier was recorded. When a compound was correctly predicted by one classifier, the compound got 1 score, or else 0 score was assigned. Finally, the total score of each compound was calculated, and the compounds with scores <2 were removed from the dataset. This reduction led to a new training set of 1254 compounds, containing 636 positives and 618 negatives (Supplementary file S2).

### 2.2. Data Analysis

**Figure 1:**
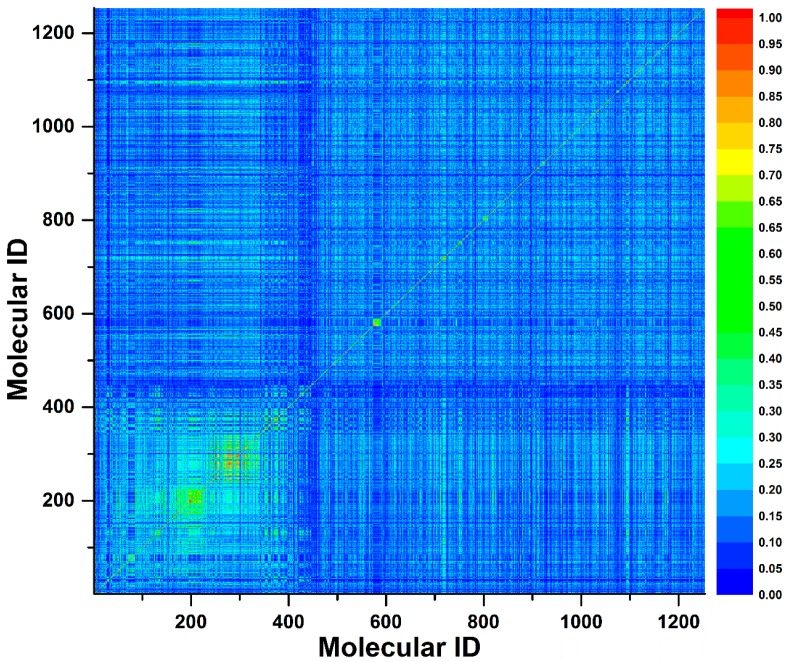
A contour graph plotted by Tanimoto similarity index to show the molecule similarity. The Tanimoto similarity index was calculated by FP2 fingerprint.

**Figure 2:**
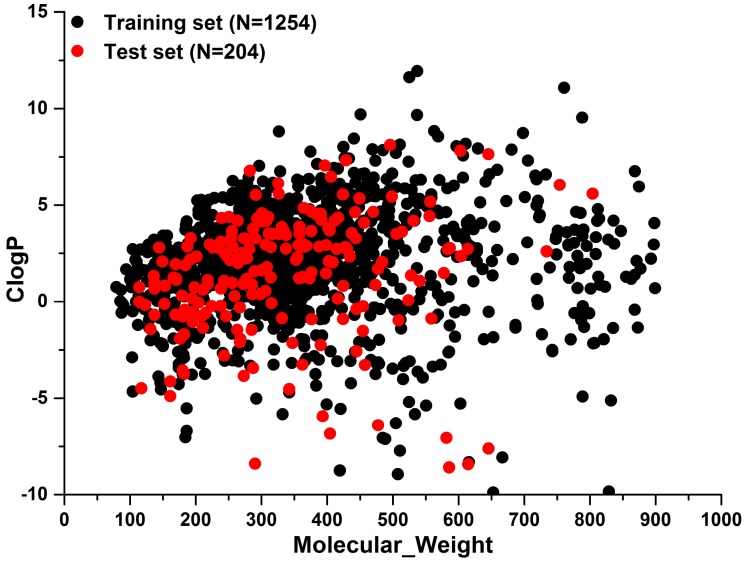
The distribution of training set and external test sets in the chemical space which was defined by molecular weight as *X*-axis and ClogP as *Y*-axis.

To investigate the chemical diversity of the modeling dataset, the Tanimoto similarity index was calculated for the whole dataset based on FP2 fingerprints. As demonstrated in Figure 1, the Tanimoto similarity indices between most compounds ranged from 0.00 to 0.30. Actually, the average Tanimoto similarity index was only 0.16, indicating the significant chemical diversity of our training set. Additionally, we investigated the chemical space of the dataset with molecular weight and calculated partition coefficient P (ClogP) as *X*-axis and *Y*-axis, respectively (Figure 2). As a result, the scale of molecular weight was between 79.10 and 899.08, and ClogP values ranged from −9.89 to 11.93. 

### 2.3. Model Construction and Evaluation

**Figure 3:**
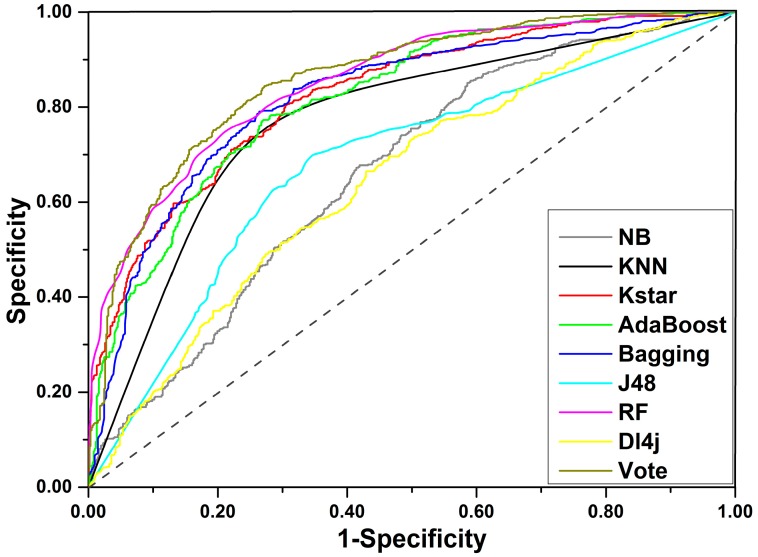
ROC curves for the training set.

For the final training set of 1254 compounds, a total of eight machine learning methods were applied to develop prediction models for DILI. As a result, eight unique models were generated. A total of five important indices, including accuracy (ACC), sensitivity (SE), specificity (SP), area under the receiver-operating characteristic curve (AUC), and balanced accuracy (BACC), were investigated to assess the performance of each model created. As listed in Table 1, ACC varied from 0.601 to 0.777, AUC varied from 0.648 to 0.852 (Receiver-operating characteristic curve (ROC) for each classifier is displayed in Figure 3), SE varied from 0.608 to 0.857, SP varied from 0.401 to 0.761, and BACC was between 0.600 and 0.777. Three out of the eight classifiers failed to yield models with acceptable performance, including Dl4j, NaiveBayes, and J48 of which the ACC values did not exceed 0.700. KNN provided the highest ACC value of 0.777 but gave a relatively lower AUC value of 0.780. For the other four classifiers, ACC and AUC were generally greater than 0.700 and 0.800, respectively. In summary, the Random Forest algorithm produced the optimal model of which ACC, SE, SP, AUC, and BACC were 0.761, 0.785, 0.736, 0.761, and 0.852, respectively. One can clearly find that the model’s performance varied with the machine learning methods, and each classifier has its own strengths and weaknesses.

Compared to single classification algorithms, ensemble algorithms always offer more stable and effective in silico models by integrating the advantages of multiple classification algorithms. For classification problems, averaging is an effective and frequently used ensemble method. Averaging takes contributions from all classifiers into consideration and is considered to be less biased than many base classifiers. Here, we attempted to integrate the eight base classifiers mentioned above to generate an ensemble model by averaging the probabilities from each classifier. As expected, all of the five indices were slightly greater than the corresponding values yield by the Random Forest algorithm. From Table 1, we can find that the ensemble model gave ACC of 0.783 and SE, SP, BACC, and AUC were 0.818, 0.748, 0.783, and 0.859, respectively. Compared to the model achieved by Random Forest classifier, ACC, SE, and SP of the ensemble model improved by 2.2%, 3.3%, and 1.2%, respectively. Therefore, we can claim that the ensemble model provided a better performance than the model derived from Random Forest classifier.

To assess the statistical significance and robustness of the ensemble model, 100 runs of Y-randomization were conducted. Consequently, the performance of Y-randomization model was rather poor with performance indicators of ACC 0.499 ± 0.018, SE 0.503 ± 0.044, SP 0.494 ± 0.037, BACC 0.498 ± 0.018, and AUC 0.499 ± 0.022. It is obvious that the ensemble model was significantly better than the Y-randomization model, indicating our model was rather robust and reliable.

### 2.4. External Validation

A total of three external datasets compiled by Ai et al. [37], Zhang et al. [30], and Kotsampasakou et al. [33] were considered in the current study (Table 2). We first compared our ensemble model with Kotsampasakou’s model of which the test set consisted of 67 molecules. As a result, prediction accuracies of our model and Kotsampasakou’s model were 67.2% and 59.7%, respectively. It is clear that our model performed better than Kotsampasakou’s model significantly. 

Zhang’s test set included 85 unique molecules. Actually, it was really a challenge for our model to compare with Zhang’s model based on this test set. One can find that Zhang’s test set consisted of 58 positives and 27 negatives. Apparently, this test set was highly imbalanced. As we know, Zhang’s model may be a biased model of which the SE and SP were 0.948 and 0.585, respectively. In other words, Zhang’s model is preferred to predict the test data as positives. Therefore, it was easy to yield acceptance prediction accuracy for Zhang’s model when the test set was dominated with positives. However, interestingly, Zhang’s model provided ACC of 68.2%, which was significantly lower than our model’s 74.1%, indicating the satisfactory predictive capability of the model developed in the current study.

As a publicly accessible prediction model for DILI, Ai’s model was developed based on 1241 diverse compounds by an ensemble learning method. Compared to many prior studies, Ai’s model showed a stronger predictive power [37]. Ai’s test set consisted of 66 positives and 17 negatives. Similar to Zhang’s test set, this test set was also dominated with positives. It was reported that Ai’s model provided a higher SE (0.799) and a lower SP (0.603). Compared to Ai’s model, our model gave a relatively unbiased performance with SE and SP of 0.818 and 0.748, respectively. It is clear that there was less challenge to predict such a positives-dominant dataset for Ai’s model. Interestingly, the performance of our model was as good as Ai’s model. As demonstrated in Table 2, the same to Ai’s model, our model also yielded an ACC value of 0.831. In addition, our model provided an SP value of 64.7%, which was higher than Ai’s model by 11.8%. However, we only correctly predicted two negatives more than Ai’s model. So large a difference of the SP value between our model and Ai’s model may be attributed to the small proportion of negatives in the test set. After all, there were only 17 negatives included in this test set. In summary, we can claim that there was no significant difference between Ai’s model and our model in the ability to detect positives. However, it was indeed difficult to answer the question of whether our model outperformed Ai’s model when predicting negatives based on such a positives-dominant dataset. To answer this question, a reverse validation was performed based on a large negative dataset. A total of 312 non-DILI molecules were included in this negative dataset. As a result, our model provided an ACC value of 68.9%, which was significantly higher than Ai’s model of 30.1%, indicating the stronger ability of our model to recognize negatives. Taken together, we can speculate that our model outperformed Ai’s model significantly. In addition, the performance of our model against the entire external test set was provided in Table 2. For the entire external test set, our model gave SE, SP, ACC, and BACC of 0.773, 0.658, 0.730, and 0.716, respectively.

These comparisons demonstrated that our model outperformed many existing prediction models, but also highlighted the deficiency of works reported in the literature.

## 3. Discussion

As one of the major causes of drug failure in clinical trials, DILI seriously impeded the development of new drugs. Although a series of in silico models for DILI have been reported during the past decades, it indeed remained a huge challenge to predict DILI successfully. One reason for such a situation may be attributed to the relatively complicated mechanism of DILI. Generally, DILI was divided into three types according to the type of liver damage and the clinical chemistry biomarker alterations, including cholestatic liver injury, hepatocellular liver injury, and mixed liver injury (hepatocellular and cholestatic) [38]. Some researchers even divided DILI into 21 hepatotoxicity endpoints [36], indicating the highly complicated and diverse mechanism of DILI. So complex a mechanism increased the difficulty in creating an in silico model for DILI. The poor quality of the dataset may be another reason for the disappointing performance of in silico models for DILI. Owing to the lack of a ‘gold standard’ that defines DILI risk, it is difficult to evaluate the accuracy of DILI annotation. Data from different sources with conflicting labels always brought noise to the training sets and decreased the accuracy of models. Theoretically, mechanism-based methods may achieve a satisfactory performance. However, currently, the hepatotoxicity mechanisms of many drugs are far from elucidated. Therefore, it may be more realistic to improve the predictive capability of in silico models for DILI by improving the quality of the datasets.

In this work, a comprehensive literature retrieval was conducted followed by a critical data filtering. Consequently, a large-scale and high-quality dataset for DILI was attained. Notably, such a large dataset for DILI was considerably rare in prior works. It should be emphasized that this dataset was not acquired by a simple integration of data provided by prior studies. According to statistics, a total of 727 unique compounds were collected from literature reviews by us. We thought the excellent performance of our model partly benefited from such a high-quality dataset. Additionally, data provided by the current study will be a valuable source for modeling/data mining in the future.

As natural products, herbs and medicinal plants are usually deemed as safe and widely used in many Asian countries. Recently, ingredients from medicinal plants have gained increasing attention for their significant efficacy in the treatment and prevention of many diseases, and played essential roles in drug discovery and development. However, natural products are not always beneficial to health. Several commonly used medicinal plants have been proven to induce liver injury [39,40]. Data provided by Drug-Induced Liver Injury Network in the United States showed that 16% of clinical hepatotoxicity cases were attributable to herbal and dietary supplements [41]. Additionally, a multicenter investigation from China reported that not less than 39% of clinical DILI were ascribed to herbs. Therefore, DILI labeling for natural products would be completely necessary. Kim et al. [24] and Huang et al. [25] have attempted to screen ingredients with DILI risks from traditional Chinese medicines (TCMs) by QSAR models created based on the Liver Toxicity Knowledge Base (LTKB) database. However, in silico models generated only by synthetic drugs often lacked applicability to natural products, which was typically attributed to inadequate coverage of the chemical space of interest. By integrating 11 herbal ingredients and the LTKB dataset, Zhao et al. [26] established a QSAR model for assessing the DILI risk of ingredients from TCMs. It turned out that adding natural hepatotoxins into the modeling dataset assists the QSAR model to provide more rational results when applied to TCMs. In the current study, we collected hundreds of hepatotoxic/nonhepatotoxic ingredients from medicinal plants and added them into the modeling dataset, increasing the size and diversity of the training set. Therefore, theoretically, compared to a model solely derived from synthetic drugs, our model would provide a better performance when assessing the DILI risk of natural products.

Many researchers always highlight that it is more significant to recognize hepatotoxins than to identify nonhepatotoxins [24,26,37]. However, it is indeed not a perfect strategy to increase the sensitivity by decreasing the specificity. After all, lower specificity always leads to a higher false positive rate, and diminishes the reliability of results acquired. Currently, many QSAR models show very limited predictability in differentiating compounds with “No-DILI” assignment, typically due to the unbalanced datasets [25,30,37]. In this work, the Kennard–Stone algorithm was utilized to achieve a balanced dataset, by which we attempted to develop an effective and unbiased QSAR model for DILI. As expected, our model yielded a considerably satisfactory SP value of 0.748, which is significantly higher than SP values (0.317–0.700) provided by many prior studies [29,30,31,32,33,34]. We also made a comparison of the ability to identify negatives between our model and Ai’s model using a large negative dataset containing 312 unique nonhepatotoxic molecules. As a result, our model outperformed Ai’s model significantly. 

Although our model outperformed many prior studies significantly, there indeed existed some limitations which were difficult to avoid. In this work, the DILI labels were only categorized into two groups: DILI-negative and DILI-positive. However, in many publications, DILI was categorized into multiple levels according to the severity of DILI risk [35,42,43,44,45]. Generally, multiple class labels lead to a more accurate QSAR model by decreasing the complexity of the characteristic pattern. However, it is difficult to find a unified criterion to define the DILI risk. The lack of sufficient data also increased the difficulty in developing a multiple-level QSAR model for DILI. In addition, similar to many prior studies, the relationship between dose and incidence of DILI was not considered in the current study. There is no doubt that dose indeed plays an important role in the occurrence of DILI. However, data provided by different laboratories always adopted different detection methods and evaluation indices. Therefore, it is actually a challenge to integrate data from different sources. Taken together, a large-scale dataset with standard annotations and dose information will help to develop a more effective QSAR model for DILI risk assessment. 

Improvements in several aspects as follows may contribute to the construction of an in silico model with stronger predictive power:

(1) In this work, all of the parameters and configurations of the machine learning algorithms were set to the default values provided by Waikato Environment for Knowledge Analysis (WEKA). The performance of our model may be improved by adopting more appropriate parameters.
(2) In the development of the ensemble model, averaging method was utilized to integrate multiple classifiers. Actually, there have been many other classifier fusion strategies: Mean, Maximum, Multiple, and so forth. In future research, comparisons among different classifier fusion strategies are needed.
(3) In addition, only 55 molecular descriptors were used in the development of the in silico model. With the development of computational chemistry, hundreds of molecular descriptors are available as well as dozens of molecular fingerprint systems. The introduction of more molecular descriptors/molecular fingerprints may contribute to acquiring an in silico model with better performance.

## 4. Materials and Methods

### 4.1. Datasets

The existence of publicly accessible datasets for DILI offers us an opportunity to gain an integrated dataset. Here, a comprehensive investigation into datasets for hepatotoxicity was performed by retrieving PubMed database with the following terms: “drug-induced hepatotoxicity”, “drug-induced liver injury”, and “DILI”. It should be pointed out that data from text mining and in silico prediction was not considered in the current study. Consequently, 14 unique datasets, which have been used in much research, were identified and are detailed in Table 3. Most of the hepatotoxicity labels for these datasets originated from animal/cell experiments, clinical reports, drug labels, medical monographs, and scientific literature.

New datasets always deserve more attention and play an essential role in data-driven science. To collect new data for hepatotoxicity, a system literature retrieval, focusing on natural products with hepatotoxicity/hepatoprotection, was performed based on PubMed and CNKI (China National Knowledge Infrastructure) database between 2009 and 2018. In Table 4, the list of search terms that were used to collect hepatotoxicity/hepaprotective compounds is displayed. As a result, a series of scientific literature was obtained. Then, we collected hepatotoxicity/hepaprotective compounds by reviewing these scientific publications one by one. To ensure the reliability of DILI risk annotation, each record was verified by two individuals simultaneously. As a result, 673 hepatotoxicity records were acquired, as well as 2914 hepatoprotective records. The compounds, without hepatotoxicity records, that showed liver protective effects were labeled as “negative”, and the compounds that induced any hepatotoxicity were flagged as “positive”.

### 4.2. Data Preprocessing

**Figure 4:**
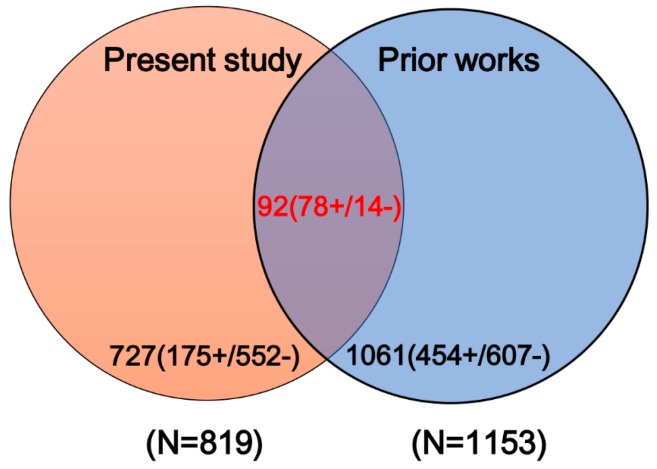
Venn diagram of compounds from prior studies and the present study. “+” and “−” denote the number of DILI-positives and DILI-negatives, respectively.

Data standardization is usually a starting point to integrate different data sources. In the present study, the data preparation procedures were detailed as follows:

(1) The simplified molecular input line entry system (SMILES) information of each compound was retrieved from PubChem Compound database by name/CAS matching.
(2) Any compounds containing metal and rare atoms were discarded, as well as inorganic compounds and mixtures.
(3) The standardizer tool (Version 18.23.0) from Marvin was used to unify the structures of compounds. (I) Salts were converted into the corresponding bases or acids; (II) water molecules and solvents were deleted; (III) all aromatic compounds were normalized to kekule form; (IV) Neutralize model was implemented to neutralize the molecules. Finally, Clear Stereo model was utilized to clear the stereo information of the molecule.
(4) Compounds containing carbon atoms fewer than 4 or with molecular weight greater than 900 were deleted from the datasets.
(5) High-quality label annotation always assists in creating accurate and reliable in silico models. To minimize the risk brought by ambiguous data, a critical data screening strategy was established. Data from DILIrank [42], Livertox [44], and LTKB [45] were retained directly and marked as dataset 1. For other datasets, the compounds included in dataset 1 were discarded, and the remaining data were named as dataset 2. Compounds with conflicting class labels were removed from dataset 2, and the remaining data were utilized to develop a large-scale dataset for hepatotoxicity in combination with dataset 1. Finally, a total of 1880 compounds were retained, 707 positives and 1173 negatives. As shown in Figure 4, a total of 1153 compounds originated from datasets of prior studies, and the present study provided 819 compounds (727 new compounds).
(6) Machine learning algorithms are known to work best on balanced datasets. Imbalanced datasets usually bring challenges to the development of in silico models, and may lead to biased performance of prediction models established [51]. To avoid the risk brought by unbalanced datasets, the Kennard–Stone algorithm was used to balance the datasets provided by prior studies and the present study. Finally, we acquired a large-scale balanced dataset for hepatotoxicity, including 707 positives and 709 negatives, which were used for the following analysis.

### 4.3. External Validation

Performance comparisons to prior studies based on publicly available external validation sets were indispensable and always deserve more attention. To evaluate the generalization ability of our models, external test sets collected by Ai et al. [37], Zhang et al. [30], and Kotsampasakou et al. [33] were used. The data preparation procedures were the same as for the training set. When we collected external test sets, we found that some compounds in the external test sets of Ai et al. and Kotsampasakou et al. appeared in their training sets. To perform a fair comparison, data included in the training sets were removed from the external test sets. It should be emphasized that all of the data included in the external test sets had been removed from our training set in the process of data preprocessing. In addition, in section data preprocessing, the Kennard–Stone algorithm was utilized to extract negatives from the original negative dataset. As a result, a total of 312 non-DILI ingredients collected by the present study were removed. In the following analysis, as a reverse validation dataset, these non-DILI ingredients were used to assess the specificity of our model. The final external test sets are listed in Table 2 and Supplementary file S2.

During the past decades, there have been continuous attempts in the development of in silico models for predicting DILI. However, it is not feasible to compare our model with every model reported in the literature. Here, three latest models with stronger predictive power were selected. Comparisons between many of those old models and these three models have been conducted, which indicated that these three models were better than or at least equal to those old models. Therefore, here, we only compared our model with these three models.

### 4.4. Molecular Descriptors

As a rapidly used chemical editor, Marvin provided a series of molecular descriptors which have been proven to be useful and effective in various QSAR research [52,53,54,55]. In this work, a total of 85 molecular descriptors offered by Marvin were calculated which can be divided into two categories, physicochemical properties (30) and topological geometry properties (55).

Physicochemical properties provided data about molecular charge, elemental composition, drug-like properties, donor/acceptor count, and partition coefficients. Information on geometry structure, ring system, and other topological structures was represented by topological geometry properties. Actually, some of these molecular descriptors, including molecular polarizability, molecular weight, ClogP, and refractivity, have been demonstrated to be effective in the development of hepatotoxicity prediction systems [56,57]. A detailed description of all molecular descriptors used in this work is available in Table S1 (Supplementary file S1).

### 4.5. Feature Selection

**Figure 5:**
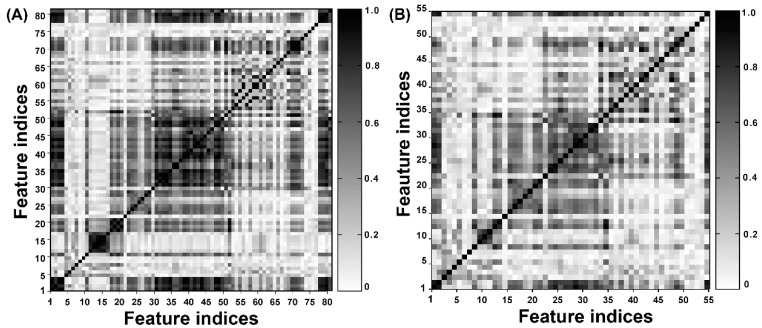
(**A**,**B**) Represents the contour graphs of the intercorrelation matrix of molecular descriptors before and after feature selection, respectively.

Redundant and irrelevant features always not only failed to assist in the development of QSAR models, but brought noise and affected the performance of in silico models significantly. Feature selection is an effective method to decrease the redundancy and degeneracy of characteristic properties [58]. In this work, molecular features, with constant values higher than 95% of all compounds, were removed. Thereafter, a correlation analysis was performed to delete the highly correlated descriptors; the cutoff value of Pearson’s correlation coefficient was set to 0.95. Finally, 55 molecular descriptors remained and are highlighted with gray in Table S1 (Supplementary file S1). From Figure 5A, one can find that a large number of highly correlated features existed in the initial feature matrix, which were removed from our defined molecular descriptors before modeling. Figure 5B shows the correlation matrix of the remaining descriptors where one can clearly find that the redundancy of the molecular features was decreased significantly.

### 4.6. Model Construction

Seven effective and widely used classifiers were adopted to create in silico models, including Naïve Bayes, K-nearest neighbor, Kstar, AdaBoostM1, Bagging, Decision tree, and Random Forest. A detailed description of these classifiers is available in Supplementary file S1. Additionally, Deeplearning4j, a deep learning classifier, was adopted. All the classification algorithms were implemented using WEKA (Waikato Environment for Knowledge Analysis, version 3.8) [59] with the default parameters and configurations, and all the classification procedures took place within 10-fold cross-validation.

### 4.7. Performance Evaluation

To investigate the predictive ability of each model developed in the current study, four important indicators were calculated, including accuracy (ACC = (TP + TN)/(TP + TN + FP + FN)), balanced accuracy (BACC = (SE + SP)/2), sensitivity (SE = TP/(TP + FN)), and specificity (SP = TN/(TN + FP)). TN, TP, FN, and FP stand for the count of true negatives, true positives, false negatives, and false positives, respectively. ACC indicates the overall classification performance. SP and SE always represent the ability to correctly recognize negatives and positives, respectively. BACC is the mean of SE and SP.

Another frequently used method in the estimation of in silico models, receiver operating characteristic (ROC) analysis, was also performed. Compared to SE and SP, which reflect the model’s performance at a single parameter, ROC analysis provides us with a more global and unbiased evaluation. Herein, area under the receiver operating characteristic curve (AUC) was calculated of which the values were between 0.5 and 1.0. A random classifier always returns AUC of 0.5. Conversely, for a perfect classifier, the AUC would reach 1.0 [60].

### 4.8. Y-Randomization

To measure the statistical significance and robustness of models developed, Y-randomization test [61] was carried out. This is done by randomly permuting the DILI annotations 100 times without alerting the ratio of positives and negatives, and leaving all descriptor data untouched. Then, we applied the same model-building procedure of the training set to the random permutation data. Ideally, the performance of Y-randomization models should be lower than the models based on real data significantly.

**Figure 6:**
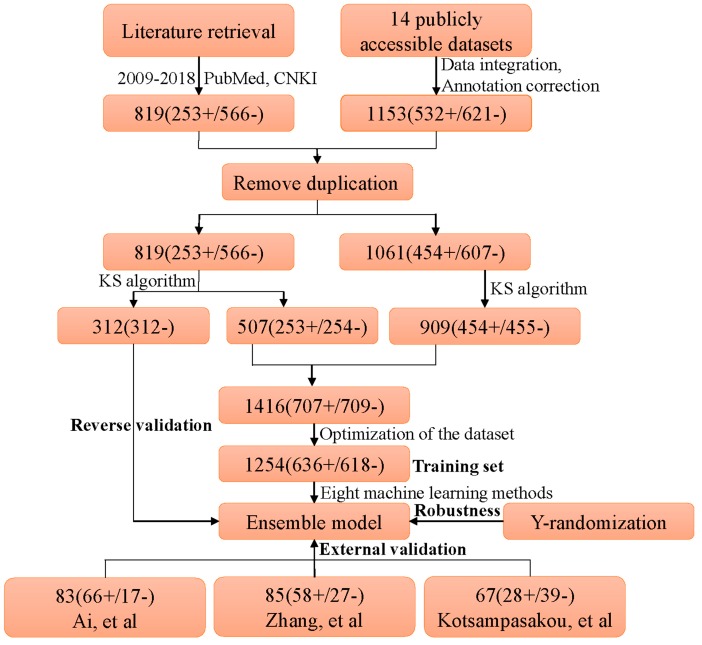
Diagram of data processing and model construction. KS algorithm: Kennard–Stone algorithm. “+” and “−” denote the number of DILI-positives and DILI-negatives, respectively.

The entire diagram of data processing and model construction is illustrated in Figure 6.

## 5. Conclusions 

In the current study, a comprehensive dataset consisting of 1254 unique compounds was built through a system literature retrieval. Then, a set of topological geometry properties and physicochemical properties were calculated with Marvin and used to train classifiers within 10-fold cross-validation. A total of eight effective and widely used classifiers were involved, including Naïve Bayes, KNN, Kstar, AdaBoostM1, Bagging, J48, Random Forest, and Dl4j. Consequently, the optimal model was attained by Random Forest algorithm of which ACC, SE, SP, AUC, and BACC were 0.761, 0.785, 0.736, 0.761, and 0.852, respectively. To improve the predictive power of our model, we integrated the eight base classifiers mentioned above to generate an ensemble model by averaging the probabilities from each classifier. As expected, the ensemble model achieved a better performance; ACC, SE, SP, BACC, and AUC were 0.783, 0.818, 0.748, 0.783, and 0.859, respectively. For further validation, comparisons between our model and prior studies were conducted based on three external test sets and a negative dataset. As a result, both the internal and external validation demonstrated that our model outperformed prior studies significantly, indicating our model was considerably successful. We believe the current work will assist in evaluating the DILI risk of drug candidates in the early stage of drug discovery.

## Figures and Tables

**Table 1:** Performances of nine models developed based on different machine learning algorithms, in terms of five different indices.

Classifier	Training Set				
	SE	SP	ACC	BACC	AUC
NaiveBayes	0.857	0.401	0.632	0.629	0.662
KNN	0.792	0.761	0.777	0.777	0.780
KStar	0.737	0.735	0.736	0.736	0.824
AdaBoostM1	0.774	0.723	0.749	0.749	0.818
Bagging	0.764	0.754	0.759	0.759	0.820
J48	0.662	0.672	0.667	0.667	0.682
Randomforest	0.785	0.736	0.761	0.761	0.852
Dl4j	0.608	0.592	0.600	0.600	0.648
Vote	0.818	0.748	0.783	0.783	0.859

**Table 2:** Comparison between our model and prior studies.

Dataset	References	Size of Dataset	SE	SP	ACC	BACC
**Training**	Present study	1254 (636+/618−)	0.818	0.748	0.783	0.783
	[37]	1241 (683+/558−)	0.799	0.603	0.711	0.701
	[30]	978 (571+/407−)	0.948	0.585	0.797	0.767
	[33]	996 (541+/455−)	0.680	0.610	0.650	0.645
**Test**	[37]	83 (66+/17−)	0.879	0.647	0.831	0.763
			(0.909)	(0.529)	(0.831)	(0.719)
	[30]	85 (58+/27−)	0.707	0.815	0.741	0.761
			(0.848)	(0.345)	(0.682)	(0.597)
	[33]	67 (28+/39−)	0.786	0.590	0.672	0.688
			(0.536)	(0.641)	(0.597)	(0.588)
	Present study (Entire external test set)	204 (125+/79−)	0.773	0.658	0.730	0.716
	Present study (Reverse validation)	312 (0+/312−)	-	-	0.689	-
					(0.301)	

In column of size of dataset, “+” and “−” denote the number of DILI-positives and DILI-negatives, respectively. For test, indicators within and outside parentheses were provided by prior studies and our model, respectively. We also investigated the performance of our model against the entire external test set which consisted of the external test sets provided by Ai et al., Zhang et al., and Kotsampasakou et al.

**Table 3:** Datasets of hepatotoxicity from prior studies.

ID	Source Name	Type of Data	No. of Compound (Positive/Negative)	DILI Categories
1	(Xu et al., 2008) [46]	Clinical data for hepatotoxicity	344 (200/144)	Authors definition
2	(Low et al., 2011) [47]	Animal experiment	127 (53/74)	Authors definition
3	(O’Brien et al., 2006) [48]	In vitro cell-based assay	83 (42/41)	Severely hepatotoxic drugs and nontoxic drugs were considered as positives and negatives, respectively
4	(Rodgers et al., 2010) [28]	Clinical data for hepatotoxicity	393 (75/318)	Actives were defined as positives, and inactives were considered as negatives
5	(Greene et al., 2010) [19]	Literature reviews and medical monographs	425 (273/152)	HH and NE represented positives and negatives, respectively
6	(Ekins et al., 2010) [23]	Clinical data for hepatotoxicity	532 (311/221)	Authors definition
7	(Liew et al., 2011) [31]	Medical monographs	1274 (759/515)	Authors definition
8	(Liu et al., 2011) [35]	Drug labeling and clinical case reports	1294 (724/570)	Authors definition
9	(Chen et al., 2013) [49]	FDA-approved drug labeling	387 (176/211)	Authors definition
10	(Zhu and Kruhlak, 2014) [50]	Postmarket safety data	282 (177/105)	Authors definition
11	(Huang et al., 2015) [25]	Scientific literature	91 (83/8)	Authors definition
12	DILIrank [42]	Drug labeling and clinical data	504 (192/312)	Only ^v^Most-DILI-Concern were considered as positives, and ^v^No-DILI-Concern were considered as negatives
13	Livertox [44]	Scientific literature and public databases	343 (119/224)	Category A and Category B were combined into positives, and Category E was considered as negatives
14	LTKB [45]	FDA-approved drug labeling	195 (113/82)	Only ^v^Most-DILI-Concern were considered as positives, and ^v^No-DILI-Concern were considered as negatives

**Table 4:** Search terms for compounds with potential hepatotoxicity or hepatoprotection.

Search Terms 1	Search Terms 2
Herbal	Hepatotoxicity
Medicinal plant	Liver Toxicity
Traditional Chinese medicine	Liver failure
	Liver injury
	Liver damage
	Hepatitis
	Liver cancer
	Liver Tumors
	Hepatocellular carcinoma
	Liver cirrhosis
	Hepatomegaly
	Liver neoplasms
	Fatty liver
	Jaundice
	Cholestasis
	Hepatoma
	Liver fibrosis
	Liver protection
	Hepatoprotective

## Supplementary Materials

Supplementary materials can be found at https://www.mdpi.com/1422-0067/20/8/1897/s1.

## Abbreviations

DILI	Drug-induced liver injury
CNKI	China National Knowledge Infrastructure
SMILES	Simplified molecular-input line-entry system
QSAR	Quantitative structure–activity relationship
ACC	Accuracy
SE	Sensitivity
SP	Specificity
AUC	Area under the receiver-operating characteristic curve
BACC	Balanced accuracy
WEKA	Waikato environment for knowledge analysis
KNN	K-nearest neighbor
J48	C4.5 decision tree
Dl4j	Deeplearning4j
ROC	Receiver operating characteristic analysis
TCMs	Traditional Chinese medicines
