# Supplementary material for: An In Silico Model for Predicting Drug-Induced Hepatotoxicity
Source: Int J Mol Sci. 2019 Apr 17;20(8):1897. doi: 10.3390/ijms20081897 (PMC6515336; doi:10.3390/ijms20081897)
Supplement: Supplementary file 1 [file ijms-20-01897-s001.zip › Supplementary file 1.pdf]

Table S1. List of 85 molecular descriptors.

Category	ID	Name	Description
Physicochemical properties	1	JCMolecularPolarizability	Molecular polarizability value
	2	JCAtomCount	The number of atoms in the molecule including hydrogens
	3	JCExactMass	The monoisotopic mass calculated from the weights of the most abundant natural isotopes of the elements
	4	JCMass	The average molecular mass calculated from the standard atomic weights
	5	JCWPC	Weight percentage of carbon elemental
	6	JCWPH	Weight percentage of hydrogen elemental
	7	JCWPN	Weight percentage of nitrogen elemental composition
	8	JCWPO	Weight percentage of oxygen elemental composition
	9	JCWPP	Weight percentage of phosphorus elemental composition
	10	JCWPS	Weight percentage of sulfur elemental composition
	11	JCWPX	Weight percentage of halogen elemental composition
	12	JCMicrospeciesCount	The number of microspecies
	13	JClogP	The octanol/water partition coefficient
	14	JClogD(pH=1.50)	The octanol-water distribution coefficient at any pH =1.50
	15	JClogD(pH=5.00)	The octanol-water distribution coefficient at any pH =5.00
	16	JClogD(pH=6.50)	The octanol-water distribution coefficient at any pH =6.50
	17	JClogD(pH=7.40)	The octanol-water distribution coefficient at any pH =7.40

18	HLB	The hydrophilic-lipophilic balance number (HLB number) measures the degree of a molecule being hydrophilic or lipophilic.
19	JCAcceptorCount	The count of molecular hydrogen bond acceptor
20	JCDonorCount	The count of molecular hydrogen bond donor
21	JCAcceptorSiteCount	Molecular hydrogen bond acceptor multiplicity
22	JCDonorSiteCount	Molecular hydrogen bond acceptor/donor multiplicity
23	JCDoubleBondStereoisomerCount	Returns the number of generated double bond stereoisomers of the input molecule.
24	JCLipinskiRuleof53of4	Returns TRUE if the molecule passes 3 of the following 4 criteria: Molweight: <= 500, LogP <= 5, Number of H-Donor atoms <= 5, Number of H-Acceptor atoms <= 10
25	JCLipinskiRuleof54of4	Returns TRUE if the molecule passes all of the following 4 criteria: Molweight: <= 500, LogP <= 5, Number of H-Donor atoms <= 5, Number of H-Acceptor atoms <= 10
26	JCLeadLikeness	When designing leadlike combinatorial libraries, care should be exercised not to exceed the following property values: Molweight: <= 450, LogD (on pH 7.4): >= -4 and <= 4, Number of Rings: <= 4, Number of Rotatable Bonds <= 10, Number of H-Donor atoms <= 5, Number of H-Acceptor atoms <= 8
27	JCGhoseFilter	Molweight: >= 160 and <= 480, Number of Atoms: >= 20 and <= 70, LogP: between -0.4 and 5.6, Refractivity: >= 40 and <=130
28	JCMueggeFilter	Molweight: >= 200 and <= 600, Number of Rings: <=7, Number of C Atoms >=5 and (Number of Atoms, Number of C Atoms-Number of H Atoms) >=2, Number of Rotatable Bonds <=15, Number of H-Donor atoms <= 5, Number of H-Acceptor atoms <= 10, LogP >= -2 and <= 5, PSA <= 150
29	JCVeberFilter	Compounds, which meet only these two criteria will have a high probability of good oral bioavailability in the rat: Number of Rotatable Bonds: <12, Polar Surface Area: <140 A²
30	JCBioavailability	Molweight <= 500, LogP <= 5, Number of H-Donor atoms <= 5, Number of H-Acceptor atoms <= 10, Number of Rotatable Bonds <= 10, Polar Surface Area <= 200, Number of Fused Aromatic Rings <= 5. This

			function works slightly different: if at least 6 from these criteria are TRUE for a molecule, then JCBioavailability returns TRUE, otherwise FALSE.
Topological geometry properties	31	JCBalabanIndex	The Balaban distance connectivity of the molecule, which is the average distance sum connectivity.
	32	JCHararyIndex	The half-sum of the off-diagonal elements of the reciprocal molecular distance matrix of the molecule.
	33	JCHyperWienerIndex	A variant of the Wiener index.
	34	JCSzegedIndex	The Szeged index extends the Wiener index for cyclic graphs by counting the number of atoms on both sides of each bond (those atoms only which are nearer to the given side of the bond than to the other), and sum these counts.
	35	JCWienerIndex	Returns the average topological atom distance (half of the sum of all atom distances) in the molecule.
	36	JCRefractivity	Returns the molar refractivity of the input molecule.
	37	JCAsymmetricAtomCount	Returns the number of asymmetric atoms (having four different ligands).
	38	JCChiralCenterCount	Returns the number of tetrahedral stereogenic centers in the input molecule.
	39	JCConnectedGraph	Returns TRUE if the input molecule graph is connected.
	40	JCDreidingEnergy	Calculates the energy related to the 3D structure (conformation) of the molecule using dreiding force field.
	41	JCHeavyAtomCount	Returns the number of heavy atoms in the molecule.
	42	JCMaximalProjectionArea	Calculates the maximum of projection areas of the conformer, based on the van der Waals radius (in Å2).
	43	JCMaximalProjectionRadius	Calculates the radius for the maximal projection area of the conformer (in Å).
	44	JCMinimalProjectionArea	Calculates the minimum of projection areas of the conformer, based on the van der Waals radius (in Å2).
	45	JCMinimalProjectionRadius	Calculates the radius for the minimal projection area of the conformer (in Å).

46	JCMolecularVolume	Calculates the van der Waals volume of the molecule (in Å3).
47	JCMaxZ	Returns the maximum z coordinate of the bounding box.
48	JCMinZ	Returns the minimum z coordinate of the bounding box.
49	JCRotatableBondCount	Returns the number of rotatable bonds in the molecule.
50	JCPlattIndex	Returns the sum of the edge degrees of a molecular graph.
51	JCRandicIndex	Returns the harmonic sum of the geometric means of the node degrees for each edge.
52	JCChainBondCount	Returns the number of chain bonds (non-ring bonds excluding bonds of hydrogen atoms).
53	JCCyclomaticNumber	Returns the smallest number of bonds which must be removed such that no circuit remains.
54	JCPSA9(pH=7.4)	Returns the polar surface area (PSA), which is formed by polar atoms of a molecule. It is a descriptor that shows good correlation with passive molecular transport through membranes, and so allows estimation of transport properties of drugs.
55	JCAliphaticRingCount	Returns the number of those rings in the molecule, which have non-aromatic bonds (SSSR based).
56	JCAliphaticRingCountOfSize(Size=3)	Returns the number of three-membered non-aliphatic rings.
57	JCAliphaticRingCountOfSize(Size=4)	Returns the number of four-membered non-aliphatic rings.
58	JCAliphaticRingCountOfSize(Size=5)	Returns the number of five-membered non-aliphatic rings.
59	JCAliphaticRingCountOfSize(Size=6)	Returns the number of six-membered non-aliphatic rings.
60	JCAromaticRingCount	Returns the number of aromatic rings in the molecule.
61	JCAromaticRingCountofSize(Size=5)	Returns the number of five-membered aromatic rings
62	JCAromaticRingCountofSize(Size=6)	Returns the number of six-membered aromatic rings
63	JCAromaticAtomCount	Returns number of atoms in the molecule having aromatic bonds.
64	JCAromaticBondCount	Returns the number of aromatic bonds in the molecule.

65	JCCarboRingCount	Returns the number of those rings in the molecule, which contain carbon atoms only.
66	JCCarboAromaticRingCount	Returns the number of heterocycles in the molecule containing carbon atoms only (SSSAR based).
67	JCFusedRingCount	Returns the number of fused rings in the molecule (having common bonds).
68	JCFusedAliphaticRingCount	Returns the number of aliphatic rings having common bonds with other rings.
69	JCFusedAromaticRingCount	Returns the number of aromatic rings having common bonds with other rings.
70	JCHeteroRingCount	Returns the number of those rings in the molecule, which contain hetero atoms.
71	JCHeteroAromaticRingCount	Returns number of aromatic heterocycles in the molecule.
72	JCLargestRingSize	Returns the size of the largest ring in the molecule.
73	JCLargestRingSystemSize	Returns the size of the largest ring system in the input molecule.
74	JCRingAtomCount	Returns number of ring atoms.
75	JCRingBondCount	Returns the number of ring bonds.
76	JCRingCount	Returns the number of rings in the molecule. This calculation is based on SSSR (Smallest Set of Smallest Rings).
77	JCRingSystemCount	Returns the number of ring systems in the input molecule.
78	JCSmallestRingSize	Returns the size of the smallest ring in the molecule.
79	JCSmallestRingSystemSize	Returns the size of the smallest ring system in the input molecule.
80	JCDominantTautomerCount	Returns the number of dominant tautomers.
81	JCTautomerCount	Returns the number of tautomers.
82	JCAliphaticAtomCount	Returns the number of atoms in the molecule having no aromatic bond (excluding hydrogens).
83	JCAliphaticBondCount	Returns the number of non-aromatic bonds in the molecule (excluding bonds of hydrogen atoms).

84	JCBondCount	Returns the number of bonds in the molecule including hydrogens.
85	JCChainAtomCount	Returns the number of chain atoms (non-ring atoms excluding hydrogens).

Model building

Naïve Bayes

Naïve Bayes, a simple probabilistic classifier which is developed depend on Bayes' theorem. Bayes' theorem can be formally expressed as follows.

$$P(A \mid B) = \frac{P(B \mid A) \times P(A)}{P(B)} \quad (1)$$

Where $P(A|B)$ and $P(B|A)$ indicate the probability of events A conditional on events B and the probability of events B conditional on events A, respectively. $P(A)$ is the probability of events A without considering events B. similarly, $P(B)$ is the probability of events B without considering events A. Naïve Bayes assumes that all features are strong independence between each other. Although such an assumption is often incorrect, Naïve Bayes classifier has been found to perform quite well in a variety of classification works. Even when the independent variables were highly correlated, Naïve Bayes classifier can also return satisfactory results [1, 2].

K-nearest neighbor

As an instance-based classification method, KNN may be the simplest classifier. The first step of KNN is to predefine the number of samples in training set closest to the new points. Generally, standard Euclidean distance is adopted to measure the distance between samples. The label of a new sample is determined by voting: the dominant category label of its k nearest neighbors is assigned to the new sample. As a non-generalizing machine learning method, KNN only stores instances from training set without generating internal models, and begins to learn only when the testing sample is coming. Therefore, when the data volume is large and high efficiency is required, KNN may be not the optimal choice. Nevertheless, KNN has been successfully applied in numerous classification problems [3, 4].

Kstar

As another instance-based classification algorithm, Kstar is developed based on K-nearest neighbor framework. Therefore, Kstar almost has the same features as KNN. The major difference between them is that they adopt different distance evaluation method. Kstar using entropy-based method to measure the distance between instances. In other words, the distance between instances was represented by the complexity of transforming one instance into another [5].

AdaBoostM1

As an ensemble learning algorithm, AdaBoost was always used in combination with many weak classifiers to improve the performance of *in silico* models. The basic idea of AdaBoost is that combining the output of each weak classifier into a weighted sum that stands for the final results of the boosted classifier. During the machine learning process, the weak classifiers are learned one by one. At each iteration, the following weak learners are preferred to in favor of those samples misclassified by the previous classifiers. Finally, the weak rules obtained by each weak classifier are combined to create a strong rule. In

this work, AdaBoost (with J48 as the weak learner), one of the best out-of-the-box classifiers, was adopted to create prediction model for DILI [6, 7].

Bagging

Bagging, short for Bootstrap aggregating, is always used to improve the accuracy and stability of weak classifiers. For Bagging, the re-sampling techniques were used to generate a series of new training sets. Based on the new training sets, multiple versions of a predictor were attained and integrated to be an aggregated predictor by averaging the output or voting. Different from AdaBoost, the weak classifiers of bagging were independent between each other. Compared to other classifiers, Bagging usually displayed a more strong generalization ability. Here, Bagging was used to develop predictive model with KNN as the weak classifier [8, 9].

Decision tree

Compared to other classification algorithms, decision tree algorithm is easier to understand and interpret. Models created based on this method can be displayed as decision trees consist of decision nodes, branches, and leaf nodes. As the most popular decision tree algorithm, C4.5 measures the splitting attribute based on a gain ratio impurity method. At every node of the decision tree, the attribute with the maximum information gain was choosed to divide the samples into subsets enriched in one class or the other. Thereafter, the C4.5 algorithm recurses on the partitioned sublists. In summary, the basic idea of decision tree is to predict the target variable based on the decision rules inferred from a large scale of dataset. [10, 11].

Random forest

As a widely used ensemble learning algorithm, Random forest algorithm runs by generating large numbers of decision trees simultaneously. Each tree in the forest is a sub-classifier, and the target variables of the test instances were confirmed by integrating the prediction results of each sub-classifier. For one test instance, the classification of each tree is regarded as one vote. Finally, we calculated the total votes and the class which achieves the maximum votes were assigned to the test instance. Random forest algorithm always offered an excellent performance and avoided the overfitting risk [12, 13].

References

1. Zhang, Z., Naive Bayes classification in R. *Ann Transl Med.* **2016,** *4*, 241.
2. Wolfson, J.; Bandyopadhyay, S.; Elidrisi, M.; Vazquez-Benitez, G.; Vock, D. M.; Musgrove, D.; Adomavicius, G.; Johnson, P. E.; O'Connor, P. J., A Naive Bayes machine learning approach to risk prediction using censored, time-to-event data. *Stat Med.* **2015,** *34*, 2941-2957.
3. Zhang, Z., Introduction to machine learning: k-nearest neighbors. *Ann Transl Med.* **2016,** *4*, 218.
4. Wang, X., A Fast Exact k-Nearest Neighbors Algorithm for High Dimensional Search Using k-Means Clustering and Triangle Inequality. *Proc Int Jt Conf Neural Netw.* **2012,** *43*, 2351-2358.
5. Cleary, J. G.; Trigg, L. E., K * : An Instance-based Learner Using an Entropic Distance Measure. *Proc.intl Conf.on Machine Learning.* **1995**, 108-114.
6. Romero, E.; Màrquez, L. s.; Carreras, X., Margin maximization with feed-forward neural networks: a comparative study with SVM and AdaBoost. *Neurocomputing.* **2004,** *57*, 313-344.
7. Schapire, R. E.; Singer, Y. Machine learning, improved boosting algorithms using confidence-rated predictions. *Machine Learning.* **1999**; *37*; 80-91.

8. Breiman, L., Bagging predictors. *Machine Learning.* **1996,** *24*, 123-140.
9. Wang, Y.; Li, Y.; Liu, X.; Pu, W.; Wang, X.; Wang, J.; Xiong, M.; Yao Shugart, Y.; Jin, L., Bagging Nearest-Neighbor Prediction independence Test: an efficient method for nonlinear dependence of two continuous variables. *Sci Rep.* **2017,** *7*, 12736.
10. Quinlan, J. R., Induction of decision trees. *Machine Learning.* **1986,** *1*, 81-106.
11. Khosravi, K.; Pham, B. T.; Chapi, K.; Shirzadi, A.; Shahabi, H.; Revhaug, I.; Prakash, I.; Tien Bui, D., A comparative assessment of decision trees algorithms for flash flood susceptibility modeling at Haraz watershed, northern Iran. *Sci Total Environ.* **2018,** *627*, 744-755.
12. Cutler, A.; Cutler, D. R.; Stevens, J. R., Random Forests. *Machine Learning.* **2004,** *45*, 157-176.
13. D Richard, C.; Edwards, T. C.; Beard, K. H.; Adele, C.; Hess, K. T.; Jacob, G.; Lawler, J. J., Random forests for classification in ecology. *Ecology.* **2007,** *88*, 2783-2792.
